# Association between dietary intake and urinary concentrations of caffeine and caffeine metabolites and elevated serum prostate-specific antigen (PSA) among men at risk for prostate cancer

**DOI:** 10.1007/s10552-025-02015-1

**Published:** 2025-07-02

**Authors:** Hongke Wu, Ming Wang, Alicia C. McDonald

**Affiliations:** 1 Department of Public Health Sciences, Pennsylvania State University College of Medicine, Hershey, USA; 2 Department of Population and Quantitative Health Sciences, Case Western Reserve University School of Medicine, 10900 Euclid Avenue, Robbins Building E240-C, Cleveland, OH 44106 USA

**Keywords:** Serum prostate-specific antigen, Caffeine, Caffeine metabolites, Prostate cancer

## Abstract

**Purpose:**

Prostatic chronic inflammation has been found to be associated with prostate cancer risk. Caffeine intake has shown to exhibit anti-inflammatory properties. The relationship between caffeine and prostatic inflammation remains not known. We examined whether dietary intake and urinary concentration of caffeine and its metabolites are associated with serum prostate-specific antigen (PSA) levels, a surrogate marker of prostatic inflammation, among prostate cancer-free men.

**Methods:**

Cancer-free men, aged ≥ 40 years, with dietary caffeine intake and serum PSA results were identified from the 2001–2010 National Health and Nutrition Examination Survey. Elevated serum PSA was based on age- and race-specific definitions. Weighted logistic regression analysis with survey sample weights was used to examine the association between dietary intake and urinary concentration of caffeine and its metabolites and elevated serum PSA.

**Results:**

There were 5,456 men included. Approximately 6.4% of them had an elevated serum PSA. Men with an elevated serum PSA (geometric mean: 63.4 mg) had statistically significantly lower dietary caffeine intake compared to men with a normal serum PSA (geometric mean: 80.9 mg) (*p* value < 0.01). After adjusting for confounders, dietary caffeine intake and urinary caffeine and its metabolites concentrations were not statistically significantly associated with elevated serum PSA.

**Conclusion:**

Men with elevated serum PSA had lower dietary caffeine intake compared to men with a normal serum PSA. However, dietary caffeine intake and urinary caffeine concentration were not associated with elevated serum PSA, after adjusting for confounders. Prospective studies that investigate relationships among caffeine intake and prostatic inflammation are warranted.

**Supplementary Information:**

The online version contains supplementary material available at 10.1007/s10552-025-02015-1.

## Introduction

Prostate cancer is the most common cancer and the second leading cause of cancer death among men in the U.S. [1]. Black race, older age, and family history of prostate cancer are well-established risk factors; however, dietary intake of caffeine and its metabolites as possible risk factors for prostate cancer remains unclear. Previous population-based studies have suggested that caffeine intake may play a role in decreasing the risk of various cancers such as breast, liver, ovarian, and skin cancers [2–5]. Possible explanations for this decreased risk have been attributed to caffeine’s anti-inflammatory and antioxidant properties that may have anti-cancer effects [6]. However, the relationship between caffeine and cancer risk have been inconsistent [3, 7]; therefore, its relationship with cancer risk remains not known.

Although the cell-based model studies have shown the protective effects of caffeine on prostate cancer, population-based studies have reported inconsistent results [8, 9]. For example, a recent meta-analysis including 16 prospective cohort studies found higher coffee consumption associated with lower prostate cancer risk (the pooled relative risk = 0.91, 95% CI 0.84, 0.98) [10]. A study conducted among men in Italy showed that men who had the highest consumption (over 3 cups of coffee per day) of caffeine had a 53% lower prostate cancer risk compared to men with the lowest consumption (0–2 cups of coffee per day) (*p* = 0.02) [8]. However, a review study reported no association between coffee consumption and prostate cancer risk [11]. A matched case–control study showed no association between caffeine consumption and prostate cancer; however, an inverse association was shown between the consumption of 11 mg to 20 mg of theobromine (a caffeine metabolite) and prostate cancer among older men ages > 67 years old [12]. Chronic inflammation has been identified as a possible risk for prostate cancer [13]. However, observational studies have not reported relationships between caffeine and its metabolites and inflammatory markers associated to prostate cancer risk.

Serum prostate-specific antigen (PSA) has been widely used for prostate cancer screening and a surrogate biomarker for prostatic inflammation [14]. Age- and race-specific serum PSA levels have been used to perform a prostate biopsy to determine a prostate cancer diagnosis [15]. Higher levels of serum PSA has been associated with prostate cancer and prostate cancer risk [16]. To our knowledge, the relationship between dietary intake and urinary concentrations of caffeine and its metabolites and serum PSA levels have not been fully evaluated. To evaluate possible relationships between caffeine and prostatic inflammation, we conducted a cross-sectional study to investigate the association between dietary intake and urinary concentrations of caffeine and its metabolites and elevated serum PSA among prostate cancer-free men.

## Methods

### Study population

The study population consisted of men, aged ≥ 40 years, who participated in the population-based National Health and Nutrition Examination Survey (NHANES), a nationally representative survey of the U.S. non-institutionalized population, from 2001 to 2010. Men were included if they had serum PSA and dietary caffeine and theobromine intake results. Theobromine was the only caffeine metabolite that was collected for dietary intake and therefore was included in the analyses. Men who had or ever were diagnosed with a cancer were excluded.

**Fig. 1:**
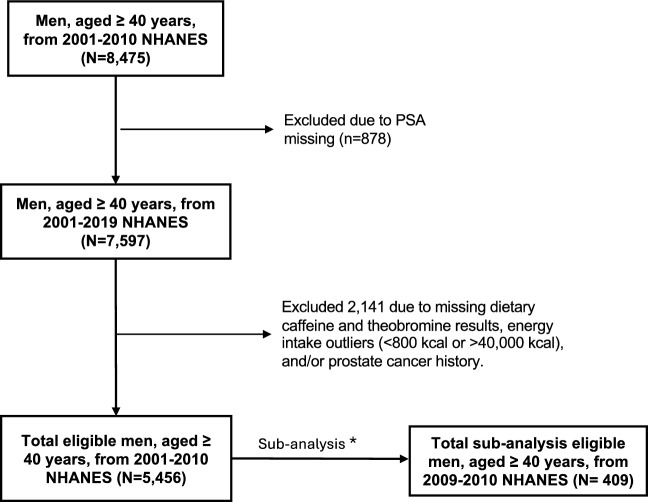
Study population flow chart

There was 8,475 men, aged ≥ 40 years, who participated in 2001–2010 NHANES of which 7,597 men had a serum PSA result. Of these men, 2,141 were excluded due to a prostate cancer or other cancer diagnosis, had a missing dietary caffeine and/or theobromine result, and/or had daily energy intake outliers. There were 5,456 men included in the final total population analyses to examine the association between dietary caffeine and theobromine intake and serum PSA levels. Out of the 5,456 men, urinary concentrations of caffeine and its 14 metabolites results were available only on men who participated in the 2009–2010 NHANES in which 409 men were eligible and included in the sub-analysis to examine the association between urinary caffeine and its metabolites concentrations and serum PSA levels. The study flowchart is shown in Fig. 1.

To participate in NHANES, informed consent was obtained from all study participants. This study was conducted according to the principles of the Declaration of Helsinki. All data were approved by the National Center for Health Statistics Research Ethics Review Board.

### Data collection

The following study variables were extracted: demographic information (i.e., age, race/ethnicity, education, and marital status), medical history (i.e., cancer diagnosis), body mass index (BMI), family history of prostate cancer, lifestyle behaviors (i.e., cigarette smoking and physical activity), dietary intake (i.e., caffeine, theobromine, water, alcohol, and energy), and laboratory measurements (i.e., creatinine, serum PSA, and urinary caffeine and its 14 metabolites). Daily dietary intake data were estimated from the type and the amount of foods and beverages consumed during the 24 hours before the interview (midnight to midnight).

Serum PSA levels were dichotomized as normal or elevated levels based on race and age-specific serum PSA cutoffs for a prostate biopsy [15]. Elevated serum PSA levels were defined as the following for white and black races by age group: (1) white men (40–49 years: > 2.5 ng/mL; 50–59 years: > 3.5 ng/mL; 60–69 years: > 4.5 ng/mL; and 70–79 years: > 6.5 ng/mL) and (2) black men (40–49 years: > 2.0 ng/mL; 50–59 years: > 4.0 ng/mL; 60–69 years: > 4.5 ng/mL; and 70–79 years: > 5.5 ng/mL) [15]. Because race-specific serum PSA was not listed for all racial/ethnic groups (i.e., Hispanic), white race’s age-specific serum PSA cutoffs were applied to the other races and Hispanic ethnicity.

### Laboratory analysis

Total serum PSA values were measured using the Access Hybritech free PSA assay [17]. Urinary creatinine concentrations were determined by using the Jaffe rate method (kinetic alkaline picrate) [18, 19]. Urinary caffeine (1,3,7-trimethylxanthine) and 14 of its metabolites (1-methyluric acid, 3-methyluric acid, 7-methyluric acid, 1,3-dimethyluric acid, 1,7-dimethyluric acid, 3,7-dimethyluric acid, 1,3,7-trimethyluric acid, 1-methylxanthine, 3-methylxanthine, 7-methylxanthine, 1,3-dimethylxanthine [theophylline], 1,7-dimethylxanthine [paraxanthine], 3,7-dimethylxanthine [theobromine], and 5-acetylamino-6-amino-3-methyluracil [AAMU]) concentrations were determined by high-performance liquid chromatography-electrospray ionization-tandem quadrupole mass spectrometry (HPLC–ESI–MS/MS) with stable isotope-labeled internal standards [20].

### Data analysis

Descriptive analyses were conducted including median with the range or 25–75 percentile for continuous variables and frequencies with proportions for categorical variables. To compare differences between men with normal and elevated serum PSA, Pearson’s *Χ*^2^ tests or Fisher’s exact tests were used for categorical variables; two-sample t-tests or Wilcoxon rank-sum tests were used for continuous variables, as appropriate. The geometric mean and standard error were calculated for daily dietary intakes (i.e., caffeine, theobromine, water, alcohol, and energy) due to their skewed frequency distribution [21].

Multivariable logistic regressions with survey sampling weights were used to determine the association between each level of dietary caffeine, dietary theobromine, urinary caffeine and its metabolites concentrations and elevated level of serum PSA in the total population and sub-population. In the total population analysis, the following sets of covariates were included for potential confounders in regression analysis: (1) continuous age, race, continuous BMI, education, smoking, daily alcohol consumption, and vigorous physical activity (Model 1) and (2) all adjusted variables in Model 1 plus daily water intake and daily energy intake (Model 2). In the sub-population analyses, in addition to covariates aforementioned, urinary creatinine was included in all of the models (Models 1–3); and, dietary caffeine and dietary theobromine, and urinary creatinine were included in Model 3. Of note, the normality of all urinary caffeine metabolites concentrations and creatinine levels was evaluated, and if violated, natural log-transformation was applied. The adjusted odds ratios (aOR) and their 95% confidence intervals (CI) were reported.

In the total population analysis, dietary caffeine and theobromine were evaluated as continuous and categorical variables. To categorize dietary caffeine and theobromine intake, 0 mg was used as the reference level and any values greater than 0 mg were categorized into tertiles: 1–99 mg, 100–242 mg, and 243–4607 mg for caffeine, and 1–19 mg, 20–70 mg, and 71–1911 mg for theobromine. Multivariable logistic regressions were applied to examine these categorical caffeine levels and elevated serum PSA in which the same confounders described in Models 1 and 2 above were adjusted for these analyses.

All hypothesis tests were two-sided with the significance level of 0.05. The analyses were performed using SAS 9.4 (Cary, NC).

## Results

Of the 5,456 men included, there were 349 men (6.4%) who had an elevated serum PSA (Table 1). The median age of the study population was 58 years. The majority of study population were non-Hispanic white men (53.19%, *N* = 2902/5456). Among men who had an elevated serum PSA, 50.72% (*N* = 177/349) and 22.06% (*N* = 77/349) were non-Hispanic white and non-Hispanic black men, respectively. Men with elevated serum PSA were of older age, less likely to participate in vigorous physical activity, and had a lower BMI compared to men with a normal serum PSA (*p* values < 0.01).

**Table 1:** Study population characteristics by serum PSA levels (2001–2010 NHANES)

Variables	Serum PSA levels (ng/mL)			*p* value
	Total Population; *N* = 5456	Normal PSA; *N* = 5107	Elevated PSA; *N* = 349	
Age in years, Median (Q1–Q3; *N*)	58 (48–69; 5456)	57 (48–68; 5107)	65 (56–74; 349)	* < 0.0001*
Age group *N* (%)				
40–49	1545 (28.32)	1493 (29.23)	52 (14.90)	* < 0.0001*
50–59	1300 (23.83)	1241 (24.30)	59 (16.91)	
60–69	1303 (23.88)	1193(23.36)	110 (31.52)	
70 +	1308 (23.97)	1180 (23.11)	128 (36.68)	
Race/ethnicity *N* (%)				
Non-Hispanic White	2902 (53.19)	2725 (53.36)	177 (50.72)	0.3357
Non-Hispanic Black	1004 (18.40)	927 (18.15)	77 (22.06)	
Hispanic	1375 (25.20)	1290 (25.26)	85 (24.36)	
Others	175 (3.21)	165 (3.23)	10 (2.87)	
Education *N* (%)				
Lower than high school	1685 (30.90)	1563 (30.62)	122 (34.96)	0.0900
High school or higher	3768 (69.10)	3541 (69.38)	227 (65.04)	
Smoking status *N* (%)				
No	2065 (37.88)	1921 (37.64)	144 (41.26)	0.1779
Yes	3387 (62.12)	3182 (62.36)	205 (58.74)	
Marital status *N* (%)				
Never been married	358 (6.57)	334 (6.54)	24 (6.92)	0.8126
Ever married	4829 (85.59)	4525 (88.66)	304 (87.61)	
Living with partner	264 (4.84)	245 (4.80)	19 (5.48)	
Vigorous physical activity (%)				
Yes	1645 (31.06)	1568 (31.59)	77 (23.12)	*0.0012*
No	3652 (68.94)	3396 (68.41)	256 (76.88)	
Body Mass Index (kg/m^2^) Median (Q1–Q3; *N*)	28.12 (25.25–31.44; 5356)	28.18 (25.30–31.53; 5018)	27.18 (24.21–30.08; 338)	*0.0028*

*PSA* Prostate-specific antigen, *N* Total number, *Q*1 First quantile, *Q*3 Third quantile

As shown in Table 2, men with elevated serum PSA had statistically significantly lower dietary intake of caffeine and energy compared to men with a normal serum PSA (*p* values < 0.003). There was no statistically significant difference in dietary theobromine, total water intake, and alcohol consumption between the two PSA groups (Table 2). After adjusting for age, race, BMI, smoke, daily alcohol consumption, and vigorous physical activity in Model 1 and with the addition of daily total energy intake and daily water intake in Model 2, inverse associations were observed between continuous (with log-transformation) and categorical (by tertile) measures of dietary caffeine intake and elevated serum PSA; these associations were not statistically significant except for continuous dietary caffeine and elevated serum PSA in Model 1 (Table 3). For both continuous and categorical dietary theobromine intake, no statistically significant associations were observed in Models 1 and 2 (Table 3).

**Table 2:** Geometric means of nutrients by serum PSA levels (2001–2010 NHANES)

Variables Geomean (Geostd)	Serum PSA levels (ng/mL)		*p* value
	Normal PSA; *N* = 5107	Elevated PSA; *N* = 349	
Caffeine (mg)	80.86 (5.76)	63.44 (6.05)	*0.0028*
Theobromine (mg)	9.18 (4.67)	8.97 (4.63)	0.8383
Water (gm)	247.43 (12.08)	203.37 (13.47)	0.2894
Energy (kcal)	2067.42 (1.42)	1925.76 (1.42)	*0.0003*
Alcohol	5.46 (3.49)	5.02 (3.29)	0.1770

*Geostd* Geometric standard deviation, *PSA* Prostate-specific antigen, *N* Total number, *gm* Gram, *mg* Milligram, *ng* Nanogram, *mL* Milliliter, *kcal* Kilocalorie

**Table 3:** Associations between dietary intake of caffeine and theobromine and elevated serum PSA levels (2001–2010 NHANES)

Variables	# Normal/Elevated PSA level	Model 1	Model 2
		aOR (95% CI)	aOR (95% CI)
Caffeine (mg)^b^	5107/349	*0.906 (0.823, 0.998)*	0.916 (0.834, 1.007)
Theobromine (mg)^b^	5107/349	1.016 (0.905, 1.140)	1.036 (0.922, 1.166)
Caffeine (mg)^a^			
0	487/37	*ref*	*ref*
1–99	1518/123	0.947 (0.548, 1.637)	0.978 (0.572,1.673)
100–242	1540/112	0.908 (0.477, 1.728)	0.955 (0.510,1.786)
243–4607	1562/77	0.580 (0.332, 1.033)	0.627 (0.360, 1.092)
Theobromine (mg)^a^			
0	2650/186	*ref*	*ref*
1–19	815/51	0.722 (0.430, 1.212)	0.736 (0.441, 1.226)
20–70	818/63	1.112 (0.707, 1.749)	1.166 (0.745, 1.826)
71–1911	824/49	0.916 (0.561, 1.496)	0.988 (0.598, 1.634)

Model 1: Adjusted for age (continuous), race, body mass index (continuous), education, smoke, daily alcohol consumption, and vigorous physical activity

Model 2: Adjusted for all variables in Model 1, plus daily energy intake and daily water intake

*aOR* Adjusted odds ratio, *CI* Confidence Interval, *ref* Reference, *mg* Milligram

^a^Variables are categorized by tertiles

^b^Variables are log-transformed

To examine the association between urinary caffeine and its 14 metabolites concentrations and elevated serum PSA, there were a total of 409 men included in the sub-analysis: 386 men with normal serum PSA group and 23 men with elevated serum PSA (Table 4). Increasing levels of 1-methyluric acid (aOR = 1.592, 95% CI 1.059, 2.392 in Model 1, aOR = 1.599, 95% CI 1.069, 2.392 in Model 2) and 7-methyluric acid (aOR = 1.277, 95% CI 1.016, 1.606 in Model 1) showed a statistically significant association with elevated serum PSA; however, these associations were no longer statistically significant after adjusting for dietary caffeine and theobromine intake in Model 3.

**Table 4:** Associations between urinary caffeine and its metabolites and elevated serum PSA levels (2009–2010 NHANES)

Caffeine and caffeine metabolites (µmol/L)*	Model 1 aOR (95% CI)	Model 2 aOR (95% CI)	Model 3 aOR (95% CI)
1-methyluric acid	*1.592 (1.059, 2.392)*	*1.599 (1.069, 2.392)*	1.529 (0.958, 2.442)
3-methyluric acid	1.372 (0.873, 2.158)	1.367 (0.856, 2.184)	1.396 (0.786, 2.478)
7-methyluric acid	*1.277 (1.016, 1.606)*	1.260 (0.995, 1.597)	1.288 (0.935, 1.775)
1,3-dimethyluric acid	1.370 (0.926, 2.026)	1.384 (0.958, 1.998)	1.304 (0.858, 1.983)
1,7-dimethyluric acid	1.156 (0.825, 1.618)	1.159 (0.832, 1.614)	1.038 (0.727, 1.482)
3,7-dimethyluric acid	1.193 (0.779, 1.827)	1.175 (0.764, 1.806)	1.168 (0.782, 1.744)
1,3,7-trimethyluric acid	1.014 (0.757, 1.358)	1.019 (0.769, 1.350)	0.901 (0.653, 1.244)
1-methylxanthine	1.302 (0.889, 1.906)	1.314 (0.899, 1.920)	1.214 (0.782, 1.884)
3-methylxanthine	1.311 (0.919, 1.869)	1.291 (0.893, 1.867)	1.359 (0.803, 2.297)
7-methylxanthine	1.230 (0.872, 1.735)	1.216 (0.874, 1.691)	1.243 (0.813, 1.899)
1,3-dimethylxanthine (theophylline)	1.217 (0.882, 1.680)	1.223 (0.891, 1.677)	1.113 (0.777, 1.595)
1,7-dimethylxanthine (paraxanthine)	1.149 (0.840, 1.573)	1.157 (0.850, 1.574)	1.047 (0.724, 1.512)
3,7-dimethylxanthine (theobromine)	1.108 (0.785, 1.563)	1.084 (0.776, 1.514)	1.066 (0.755, 1.503)
1,3,7-trimethylxanthine (caffeine)	1.074 (0.819, 1.409)	1.076 (0.816, 1.421)	0.974 (0.742, 1.279)
AAMU, 5-acetylamino-6-amino-3-methyluracil	1.150 (0.799, 1.656)	1.165 (0.836, 1.623)	1.041 (0.639, 1.695)

Model 1: Adjusted for age (continuous), log-transformed creatinine, race, body mass index, education, smoking, vigorous physical activity, and daily alcohol consumption

Model 2: Adjusted for all variables in Model 1, daily energy intake, and daily water intake

Model 3: Adjusted for all variables in Model 2, log-transformed dietary caffeine, and log-transformed dietary theobromine

*aOR* Adjusted Odds Ratio, *CI* Confidence Interval, *μmol* micromole, *L* Liter

*Variables are log-transformed

The correlations among urinary caffeine metabolites concentrations and dietary caffeine and theobromine intake were examined as shown in Supplementary Table 1. Based on Dancey and Reddy’s correlation coefficient classification (poor, moderate, and strong correlations) [22], the correlation between dietary caffeine intake and urinary caffeine concentration was of moderate strength (*r* = 0.495, *p* value < 0.0001), while the correlation between dietary theobromine and urinary theobromine levels was of poor strength (*r* = 0.358, *p* value < 0.0001). There were several statistically significant positive strong correlations found among urinary caffeine metabolites (*r* ≥ 7, *p* value < 0.0001).

## Discussion

In this large, population-based study, we found that men with elevated serum PSA had a lower dietary intake of caffeine compared to men with normal serum PSA. When factors such as age, race, BMI, education, smoking, alcohol consumption, and physical activity were considered, this association was no longer statistically significant. Nevertheless, the direction of these associations may still be meaningful due to the strong magnitude of the inverse association observed between the higher levels of dietary caffeine intake (≥ 243 mg) and elevated serum PSA. The data may suggest that men who consume less caffeine may not receive the full benefits from caffeine’s antioxidant and anti-inflammatory effects which may play a role in decreasing prostatic inflammation and subsequently reducing prostate cancer risk.

Caffeine is a natural stimulant commonly found in various foods and beverages including coffee, tea, soft drinks, and energy drinks [23]. Studies suggest that caffeine has antioxidative and anti-inflammatory properties which may play a role in reducing cancer risk including prostate cancer by suppressing cell proliferation and inducing apoptosis through several oncogenic pathways, including PTEN, PI3 K/Akt, p53, and mTOR pathways [24, 25]. In the present study, men with elevated serum PSA had a lower dietary caffeine intake compared to men with normal serum PSA. After adjusting for confounders, there were no statistically significant associations observed between dietary caffeine intake and elevated serum PSA. Nevertheless, a stronger inverse association was observed at the highest tertile caffeine level (≥ 243 mg). These inverse associations may be supported by studies that found inverse relationships between coffee consumption and prostate cancer risk [8, 10].These inverse associations may imply that men who consume less caffeine may not receive the full benefits of caffeine’s antioxidative and anti-inflammatory effects compared to men who consume more caffeine as part of their diet.

Theobromine, a caffeine metabolite, is an alkaloid found primarily in cocoa beans and chocolate products [26]; and, it is present in smaller amounts in tea leaves and certain plants like the kola nut and guarana berries [26, 27]. Compared to caffeine, theobromine has milder stimulant effects; and, it has a longer mean half-life in human body [28]. Studies have shown theobromine having anti-tumor and anti-inflammatory effects in cell-based models [22, 29]. To our knowledge, there hasn’t been any observational study that evaluated the association between dietary theobromine intake and serum PSA levels. In the present study, there was no statistically significant difference in dietary theobromine intake between men with elevated and normal serum PSA.

In this present study, urinary caffeine and its metabolites concentrations were not associated with elevated serum PSA. To our knowledge, this is the first population-based study that examined urinary caffeine and its metabolites association with serum PSA. The present study is consistent with other studies that found urinary caffeine and its metabolites having no statistically significant association with other health conditions such as stroke and depression [30, 31]. A possible reason for the lack of association could be that urinary caffeine and its metabolites levels are not an accurate measurement of their dietary intake. The mean half-life of caffeine in the human body is 3–7 hours [32, 33]. For theobromine, the mean half-life is longer than caffeine, an estimated 7–12 hours in the blood [24, 28]. As a result, urinary caffeine concentrations and its metabolites may not provide measurements of long-term caffeine exposure in order to evaluate persistent caffeine exposure. This persistent exposure may play a role in reducing prostate cancer risk. Based on previous studies, dietary caffeine intake has shown to be highly correlated to urinary caffeine concentration [34]; however, this high correlation may be due to the immediate collection of urine to examine caffeine concentration levels. Studies have suggested that the concentration of caffeine and its metabolites in urine may estimate recent caffeine consumption [34–36]. In the present study, a moderate correlation between dietary caffeine and urinary caffeine and a poor correlation between dietary theobromine and urinary theobromine were observed. One possible reason for the present study findings could be that urinary samples were not collected immediately after dietary caffeine intake to measure their urine concentration, therefore, demonstrating poor to moderate correlations between dietary intake and urinary concentrations of caffeine and theobromine.

A strength of this large, population-based study is that it examined both dietary and urinary caffeine’s relationship with serum PSA among men at risk for prostate cancer. However, there were limitations to this study. Because this study was a cross-sectional study, causal relationships were not examined between dietary caffeine intake and elevated serum PSA risk. Another limitation was that a 24-hour dietary recall questionnaire was used which may have introduced recall bias; in addition, the questionnaire did not account for seasonal dietary changes of the NHANES participant. A third limitation was that there were missing data, in particular, for urinary caffeine and its metabolites concentrations which were only available for a subset of the population resulting in a smaller sample size. A fourth limitation was that NHANES participants represent a generally healthier population therefore limiting the ability to examine caffeine and its metabolites association with prostate cancer. A fifth limitation was that NHANES did not provide information on the timing of urinary sample collection after caffeine and theobromine consumption which may not reflect their total measurement in urine due to their short half-life. The timing of urine collection may explain the poor to moderate correlations observed between dietary intake and urinary concentrations of caffeine and theobromine in this study. Finally, potential confounders such as micronutrient intake were not collected and adjusted for in the analysis which could have influence the study’s associations.

In conclusion, prostate cancer-free men with elevated serum PSA consumed lower levels of caffeine compared to men with normal serum PSA in this study population. However, statistically significant associations for both dietary caffeine intake and urinary caffeine concentrations with elevated serum PSA were not observed. Nevertheless, based on the strength of the association at a higher caffeine level, these inverse associations may still provide insights on caffeine possible anti-inflammatory and antioxidant effects on prostatic inflammation which may influence serum PSA levels. To confirm these inverse relationships, prospective studies examining caffeine’s effect on prostatic inflammation and subsequent prostate cancer risk are warranted.

## Supplementary Information

Below is the link to the electronic supplementary material.

Supplementary file1 (DOCX 28 KB)

## Data Availability

Data described in the manuscript, code book, and analytic code will be made available upon request pending application and approval.
