# Supplementary material for: Association between dietary intake and urinary concentrations of caffeine and caffeine metabolites and elevated serum prostate-specific antigen (PSA) among men at risk for prostate cancer
Source: Cancer Causes Control. 2025 Jul 2;36(11):1409–16. doi: 10.1007/s10552-025-02015-1 (PMC12578734; doi:10.1007/s10552-025-02015-1)
Supplement: Supplementary file 1 — Supplementary file1 (DOCX 28 KB) [file 10552_2025_2015_MOESM1_ESM.docx]

Association between dietary intake and urinary concentrations of caffeine and caffeine metabolites and elevated serum prostatic specific antigen (PSA) among men at risk for prostate cancer

Hongke Wu, MD, MPH^1^, Ming Wang, PhD^1.2^, Alicia C. McDonald, PhD, MPH^1,2^

^1^Department of Public Health Sciences, Pennsylvania State University College of Medicine,

^2^Penn State Cancer Institute, Hershey, Pennsylvania 17033 USA

**Journal:** Cancer Causes & Control

**Corresponding Authors:**

Alicia C. McDonald, PhD, MPH

Department of Public Health Sciences

Pennsylvania State University College of Medicine

500 University Drive

Hershey, Pennsylvania 17033-0850

Email: Amcdona_97@comcast.net

Ming Wang, PhD

Department of Population and Quantitative Health Sciences

Case Western Reserve University School of Medicine

10900 Euclid Avenue

Robbins Building E240-C

Cleveland, Ohio 44106

Phone: 216-368-4526

Email: [mxw827@case.edu](mailto:mxw827@case.edu)

**Supplemental Table 1. Correlations among dietary caffeine and theobromine intakes and urinary caffeine metabolites concentrations (2009-2010 NHANES)**

|  |  | Dietary | | Urinary | | | | | | | | | | | | | | |
| --- | --- | --- | --- | --- | --- | --- | --- | --- | --- | --- | --- | --- | --- | --- | --- | --- | --- | --- |
|  | Variables,  R^2 (p-value) | Caffeine | Theobromine | 1-methyluric acid | 3-methyluric acid | 7-methyluric acid | 1,3-dimethyluric acid | 1,7-dimethyluric acid | 3,7-dimethyluric acid | 1,3,7-trimethyluric acid | 1-methylx  anthine | 3-methylx  anthine | 7-methylx  anthine | theophylline | paraxanthine | theobromine | caffeine | AAMU |
| Dietary | Caffeine | 1 | 0.204  (<.0001) | **0.511**  **(<.0001)** | 0.303 (<.0001) | 0.297  (<.0001) | **0.520**  **(<.0001)** | **0.502**  **(<.0001)** | 0.276 (<.0001) | 0.452 (<.0001) | 0.516  (<.0001) | 0.307  (<.0001) | 0.309  (<.0001) | **0.543 (<.0001)** | **0.546 (<.0001)** | 0.314 (<.0001) | 0.495 (<.0001) | **0.535**  **(<.0001)** |
|  | Theobromine | - | 1 | 0.117 (0.0178) | 0.364  (<.0001) | 0.395  (<.0001) | 0.065  (0.188) | 0.062  (0.2107) | 0.363 (<.0001) | 0.024  (0.635) | 0.078  (0.1142) | 0.388  (<.0001) | 0.396  (<.0001) | 0.013  (0.7939) | 0.012 (0.8117) | 0.358 (<.0001) | 0.006  (0.8984 ) | 0.056  (0.2626) |
| Urinary | 1-methyluric acid |  | - | 1 | **0.661 (<.0001)** | **0.675 (<.0001)** | **0.923**  **(<.0001)** | **0.889**  **(<.0001)** | **0.641**  **(<.0001)** | **0.797 (<.0001)** | **0.936**  **(<.0001)** | **0.649**  **(<.0001)** | **0.680**  **(<.0001)** | **0.742 (<.0001)** | **0.757 (<.0001)** | **0.517 (<.0001)** | **0.661 (<.0001)** | **0.831 (<.0001)** |
|  | 3-methyluric acid |  |  |  | 1 | **0.905**  **(<.0001)** | **0.642**  **(<.0001)** | **0.588**  **(<.0001)** | **0.862**  **(<.0001)** | 0.488 (<.0001) | **0.546**  **(<.0001)** | **0.934**  **(<.0001)** | **0.891**  **(<.0001)** | 0.426 (<.0001) | 0.357 (<.0001) | **0.698 (<.0001)** | 0.349 (<.0001) | **0.565 (<.0001)** |
|  | 7-methyluric acid |  |  |  |  | 1 | **0.597**  **(<.0001)** | **0.582**  **(<.0001)** | **0.883 (<.0001)** | 0.443 (<.0001) | **0.540**  **(<.0001)** | **0.927**  **(<.0001)** | **0.918**  **(<.0001)** | 0.408 (<.0001) | 0.390 (<.0001) | **0.769 (<.0001**) | 0.298 (<.0001) | **0.569**  **(<.0001)** |
|  | 1,3-dimethyluric acid |  |  |  |  |  | 1 | **0.948**  **(<.0001)** | **0.648 (<.0001)** | **0.882 (<.0001)** | **0.908**  **(<.0001)** | **0.628**  **(<.0001)** | **0.623**  **(<.0001)** | **0.859 (<.0001)** | **0.811 (<.0001)** | **0.525 (<.0001)** | **0.729 (<.0001)** | **0.896 (<.0001)** |
|  | 1,7-dimethyluric acid |  |  |  |  |  |  | 1 | **0.596 (<.0001)** | **0.922 (<.0001)** | **0.877**  **(<.0001)** | **0.612**  **(<.0001)** | **0.567**  **(<.0001)** | **0.887 (<.0001)** | **0.839 (<.0001)** | **0.542 (<.0001)** | **0.811 (<.0001)** | **0.872 (<.0001)** |
|  | 3,7-dimethyluric acid |  |  |  |  |  |  |  | 1 | **0.533 (<.0001)** | **0.596**  **(<.0001)** | **0.902**  **(<.0001)** | **0.930**  **(<.0001)** | **0.494 (<.0001)** | **0.476 (<.0001)** | **0.858 (<.0001)** | 0.361 (<.0001) | **0.557**  **(<.0001)** |
|  | 1,3,7-trimethyluric acid |  |  |  |  |  |  |  |  | 1 | **0.830**  **(<.0001)** | 0.486  (<.0001) | 0.456  (<.0001) | **0.873 (<.0001)** | **0.850 (<.0001)** | 0.501  (<.0001) | **0.903 (<.0001)** | **0.755**  **(<.0001)** |
|  | 1methylx  anthine |  |  |  |  |  |  |  |  |  | 1 | **0.573**  **(<.0001)** | **0.620**  **(<.0001)** | **0.801 (<.0001)** | **0.827 (<.0001)** | **0.517 (<.0001)** | **0.717 (<.0001)** | **0.772**  **(<.0001)** |
|  | 3-methylx  anthine |  |  |  |  |  |  |  |  |  |  | 1 | **0.957**  **(<.0001)** | 0.472 (<.0001) | 0.412 (<.0001) | **0.820 (<.0001)** | 0.332 (<.0001) | **0.578 (<.0001)** |
|  | 7-methylx  anthine |  |  |  |  |  |  |  |  |  |  |  | 1 | 0.445  (<.0001) | 0.440 (<.0001) | **0.826**  **(<.0001)** | 0.313 (<.0001) | **0.585**  **(<.0001)** |
|  | theophylline |  |  |  |  |  |  |  |  |  |  |  |  | 1 | **0.950 (<.0001)** | **0.596**  **(<.0001)** | **0.858 (<.0001)** | **0.757 (<.0001)** |
|  | paraxanthine |  |  |  |  |  |  |  |  |  |  |  |  |  | 1 | **0.596 (<.0001)** | **0.842 (<.0001)** | **0.735**  **(<.0001)** |
|  | theobromine |  |  |  |  |  |  |  |  |  |  |  |  |  |  | 1 | **0.470 (<.0001)** | **0.445**  **(<.0001)** |
|  | caffeine |  |  |  |  |  |  |  |  |  |  |  |  |  |  |  | 1 | **0.615**  **(<.0001)** |
|  | AAMU |  |  |  |  |  |  |  |  |  |  |  |  |  |  |  |  | 1 |
|  | Note: All variables are log-transformed; bolded values are significantly correlated  AAMU: 5-acetylamino-6-amino-3-methyluracil  The unit of dietary caffeine and dietary theobromine is milligram per day; The unit for urinary variable is umol/L | | | | | | | | | | | | | | | | | |
